# *CellFateScout* – a bioinformatics tool for elucidating small molecule signaling pathways that drive cells in a specific direction

**DOI:** 10.1186/1478-811X-11-85

**Published:** 2013-11-08

**Authors:** Marcin Siatkowski, Volkmar Liebscher, Georg Fuellen

**Affiliations:** 1 Institute for Biostatistics and Informatics in Medicine and Ageing Research, University of Rostock, Ernst Heydemann Strasse 8, D-18057 Rostock, Germany; 2 DZNE, German Center for Neurodegenerative Disorders, Gehlsheimer Strasse 20, D-18147 Rostock, Germany; 3 Institute for Mathematics and Computer Science, University of Greifswald, Jahnstr. 15a, D-17487 Greifswald, Germany

**Keywords:** Small molecules, Drug repositioning, Cell transformation, Induction of pluripotency, Cell fate, Bioinformatics tool, Pathway database

## Abstract

**Background:**

Small molecule effects can be represented by active signaling pathways within functional networks. Identifying these can help to design new strategies to utilize known small molecules, e.g. to trigger specific cellular transformations or to reposition known drugs.

**Results:**

We developed *CellFateScout* that uses the method of *Latent Variables* to turn differential high-throughput expression data and a functional network into a list of active signaling pathways. Applying it to *Connectivity Map* data, i.e., differential expression data describing small molecule effects, we then generated a *Human Small Molecule Mechanisms Database*. Finally, using a list of active signaling pathways as query, a similarity search can identify small molecules from the database that may trigger these pathways. We validated our approach systematically, using expression data of small molecule perturbations, yielding better predictions than popular bioinformatics tools.

**Conclusions:**

*CellFateScout* can be used to select small molecules for their desired effects. The *CellFateScout* Cytoscape plugin, a tutorial and the *Human Small Molecule Mechanisms Database* are available at https://sourceforge.net/projects/cellfatescout/ under LGPLv2 license.

## Lay abstract

Searching for chemical substances (often called small molecules) that trigger a desired effect in cells is frequently done in the laboratories of pharma and biotech companies. Computational work enables a speed-up of this search, based on three ingredients: (1) high-throughput data describing the desired effect of turning cells of type A into cells of type B, e.g. by measuring gene or protein amounts in diseased cells and in healthy cells, (2) high-throughput data measuring how such amounts change in cells if small molecules are applied, and (3) knowledge about possibly relevant cellular signaling pathways. Our procedure contributes towards the improvement of methods for finding small molecules that may turn, e.g., diseased cells into healthy cells, based on such data. The suggested small molecules can then be directly tested with a higher chance of success.

## Background

Over the last decades, drug discovery and development was the focus of many studies. Although advances were made, traditional drug development is still time-consuming, risky and costly [1]. An alternative drug discovery strategy, called repositioning, is to explore known drugs for new applications [2]. Often, repositioning is done using mechanistic knowledge of the targets and the pathways affected by the drug (small molecule), or by comparing cellular or clinical phenotypes. These approaches can be improved by computational analyses. They can aid in generating, evaluating and prioritizing data for many small molecules, targets, pathways and phenotypes simultaneously [3]. Thus, there is a rising interest in bioinformatics tools elucidating new areas of application for known small molecules, which modify, in one way or another, the state of cells, organs and organisms. Further, the repositioning paradigm reaches far beyond drug treatment. Many bioinformatics-based tools for repositioning can equally well be used to find small molecules that trigger desired cell (de-)differentiation or transdifferentiation events, such as the induction of pluripotency, endodermal or neural fate [4,5]. Data from this application area of repositioning will be used to demonstrate and validate our work, motivating the name of the tool we developed.

Various network-based methods have been developed for identifying gene expression signatures, i.e., sets of genes combined with a pattern of their expression that is typical for some cellular state. These methods have been applied e.g. to the prediction of response to drug treatment [6]. Bioinformatics network analysis of high-throughput data sets offers an opportunity for data integration considering biological complexity and multilevel connectivity [7], on the network, subnetwork, pathway or single gene/protein interaction level. Some of these network-based methods have been released as bioinformatics tools. A pioneer method that searches for so-called active subnetworks through a permutation-based algorithm is presented in [8]. It combines differential expression based p-values with a given network and is implemented by the jActiveModules Cytoscape plugin. This tool is dependent on a method that provides p-values for the change in gene expression, e.g. a t-test. It scores subnetworks by comparing two (or more) conditions, e.g. case and control. The score of a subnetwork is calculated by aggregating the z-scores of the individual genes based on the p-values; random resampling is used to estimate whether the subnetwork score is higher than expected by chance. To find the highest-scoring subnetwork in a full network, jActiveModules uses a heuristic approach based on simulated annealing. jActiveModules utilizes the network structure to find a connected set of genes, however it is not taking into account whether the considered network region is highly connected. Also, it does not consider the directionality of links. While jActiveModules is performing subnetwork evaluation based on calculating genewise p-values, another network based bioinformatics tool, KeyPathwayMiner, requires the explicit selection of significantly differentially expressed genes. Based on this information, KeyPathwayMiner finds highly connected subnetworks where most genes are expressed in most samples [9]. This bioinformatics tool, just as jActiveModules, depends on another method, which classifies genes as significantly differentially expressed. Like jActiveModules, KeyPathwayMiner is also able to compare two or more conditions. The method considers the connectivity in a network, assuming that active regions consist of differentially expressed genes that are highly connected. However, link directionality is not considered either. According to [9], KeyPathwayMiner outcompeted CUSP [10], GiGA [11], jActiveModules and a standard t-test. Another bioinformatics tool that is employed for unveiling network activity is ExprEssence [12]. Here, the authors developed a method inspired by the law of mass action that highlights interactions (links) for which the two connected genes/proteins feature a large amount of coordinated change. This method takes link directionality into account; every link can be specified as an inhibition, stimulation or interaction between two genes. ExprEssence is focused on finding single links connecting genes with coordinated change of expression, thus, an entire subnetwork is highlighted only as appropriate, that is, only if all links show high expression change. ExprEssence does not consider issues of statistical significance; it just returns a list of links, sorted by a score reflecting differential change. All mentioned tools, jActiveModules, KeyPathwayMiner and ExprEssence, are based on easy to interpret models that are computationally fast. Users of these approaches usually assume that the signaling between proteins is reflected by gene expression change.

**Figure 1:**
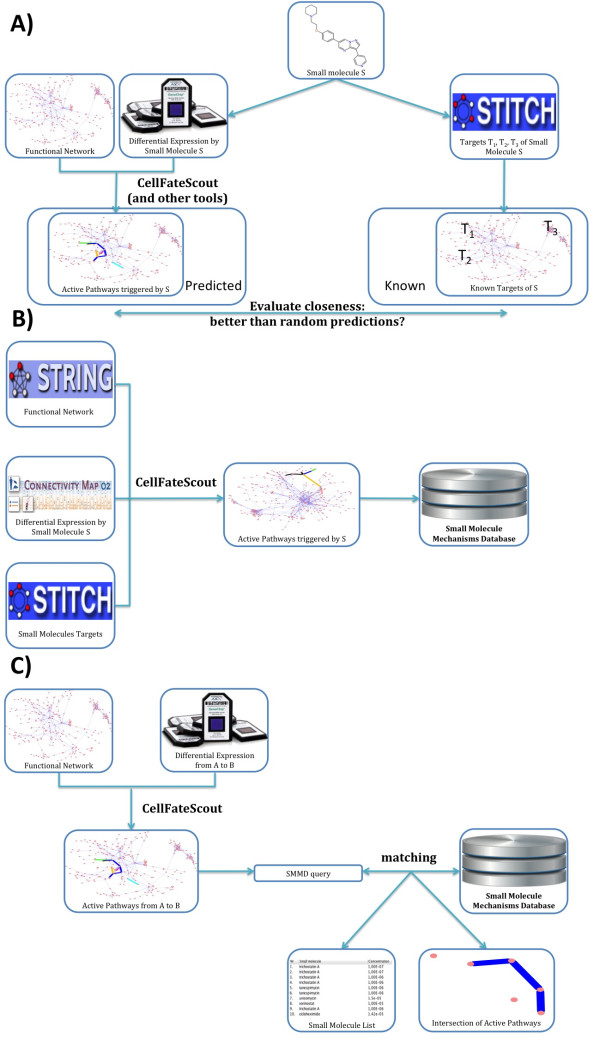
**CellFateScout study workflow. A)** Validation pipeline. For each small molecule considered, we collected publicly available differential gene expression data describing its effect, information about its targets in STITCH, and a functional network. Using CellFateScout and other bioinformatics tools, we predicted the signaling pathways that it activates and evaluated how much closer these are to its known targets in the network, compared to randomly generated pathways. **B)** We combined gene expression data taken from the Connectivity Map project, information about small molecules targets from STITCH and functional networks from STRING to perform a systematic search with CellFateScout for active pathways triggered by small molecules and stored them in the Small Molecule Mechanisms Database. **C)** As input for active signaling pathway discovery, a functional network and a differential expression data set featuring two conditions **A** and **B** are needed. The discovered active pathways are shown in a Cytoscape network view and used to query the Small Molecule Mechanisms Database for matching signaling pathways triggered by small molecules. The resulting small molecules are then listed in a result table and we can highlight edges and nodes that are shared between the active pathways found by CellFateScout and the active pathways triggered by small molecules.

In this study, we developed a network-based bioinformatics tool and Cytoscape plugin called *CellFateScout* that explores an expression signature in a network context and then suggests the repositioning of small molecules to emulate the given signature. The workflow of our study is illustrated in Figure 1. In our approach, a signature is defined by a set of pathways in the form of linear chains, representing a set of active signaling pathways in the network. For finding such a signature, we utilize the flexibility of the Latent Variable Model framework [13,14]. This flexibility allowed the authors of [15] to use latent variables for biological network analysis in a different context. We demonstrate that the Latent Variable Model approach is able to predict small molecule effects in terms of expression signatures better than other bioinformatics tools. We employ genome-wide expression profiles of cells treated with small molecules from the Connectivity Map [16]. We use CellFateScout to find the active signaling pathways based on the expression signatures of these small molecules, and store them in the Human Small Molecule Mechanisms Database (SMMD). Having a tool that can reveal active signaling pathways from a high throughput expression experiment, we can finally identify small molecules from the SMMD that may emulate the signaling mechanisms underlying that experiment.

## Results and discussion

In this study, we introduce a bioinformatics tool called CellFateScout that is implemented as a Cytoscape plugin. Using the method of Latent Variables [13,14], our tool reveals active signaling pathways based on differential expression data from two conditions, interpreted in the context of a functional network.

### Validation of CellFateScout

We performed a validation of our method against four other bioinformatics tools answering the question: how far away from the known targets of the small molecules are the active pathways we find? In [14], the performance of the Latent Variable Model is illustrated by a set of simulation studies and an analysis of gene expression data for environmental stress response in yeast. Here, we perform a detailed and systematic analysis of their approach as implemented by our plugin, including a comparison of CellFateScout with other available and well-known bioinformatics tools, representing distinct methods for elucidating pathway information from expression data. From the tools mentioned in the introduction, we compare to jActiveModules, KeyPathwayMiner and ExprEssence. Often, changes in expression levels on a genome-wide scale are still assessed on an individual basis, essentially resulting in long lists of genes that are found to have significant change, and sometimes the simplest approaches (as well as random guessing of results) may outperform complicated approaches. Therefore, we also compare to the limma [17] package from Bioconductor [18], simply selecting the most differentially expressed genes as pathway elements activated by a small molecule. CellFateScout and the other tools were subjected to the same validation protocol and the resulting p-values were computed in the same manner. This allowed a systematic and comprehensive comparison of all tools. The workflow of our validation is illustrated in Figure 1A. The selection of small molecules, corresponding gene expression data and functional networks as well as the definition of target neighborhoods and of the distance between the known targets and the gene sets found by a bioinformatics method are described in ‘Methods’.

**Figure 2:**
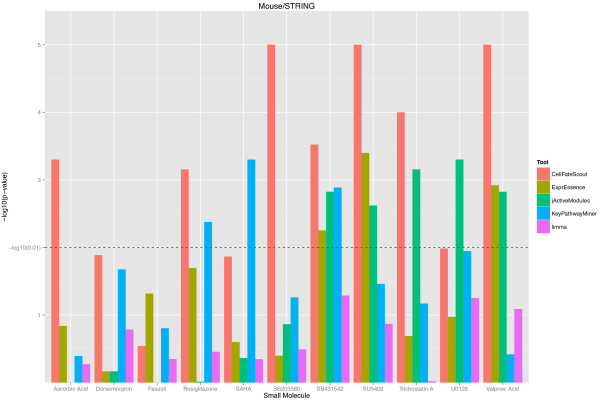
**CellFateScout validation using mouse data and STRING networks.** The histograms of -log10 transformed p-values are shown for 11 small molecules of Table 1, for 5 different bioinformatics tools. p-values indicate how much better the pathways calculated by the various tools perform in terms of their proximity to the known small molecule targets, compared to randomly generated pathways of same size. Higher histogram bars indicate superior performance, i.e., the pathways found by the bioinformatics tool are closer to the known targets.

**Figure 3:**
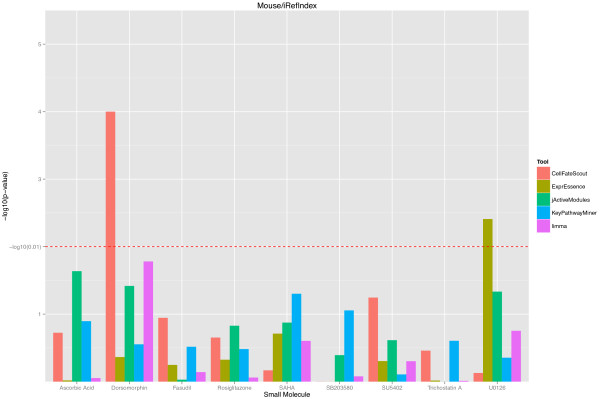
**CellFateScout validation using mouse data and iRefIndex networks.** The histograms of –log10 transformed p-values are shown for 9 small molecules of Table 1, for 5 different bioinformatics tools. See the Figure 2 legend for details.

**Figure 4:**
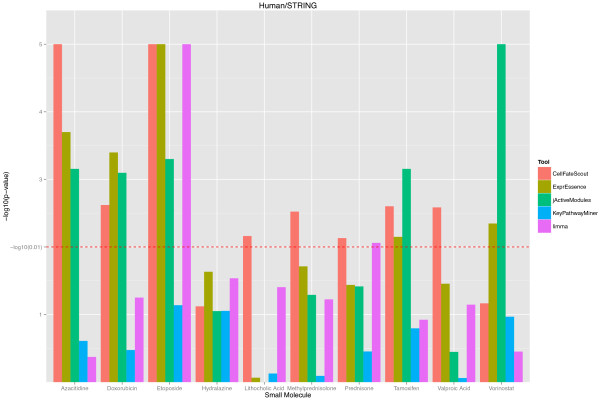
**CellFateScout validation using human data and STRING networks.** The histograms of -log10 transformed p-values are shown for 10 small molecules of Table 2, for 5 different bioinformatics tools. See the Figure 2 legend for details.

**Figure 5:**
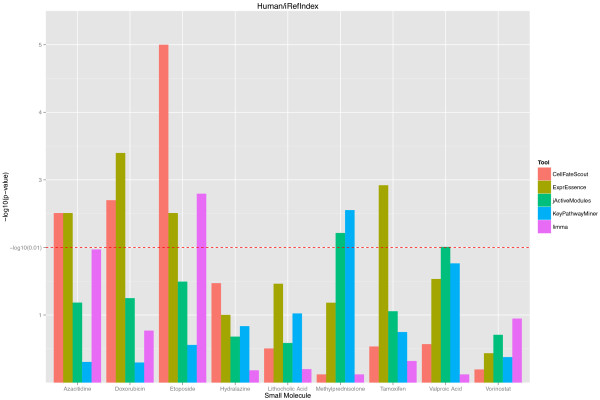
**CellFateScout validation using human data and iRefIndex networks.** The histogram of –log10 transformed p-values are shown for 9 small molecules of Table 2, for 5 different bioinformatics tools. See the Figure 2 legend for details.

Based on our validation protocol, we obtained p-values describing the closeness of the known targets of selected small molecules to the active subnetworks/pathways estimated by bioinformatics. For better visualization, we turned the p-values into histogram bar heights, where large height corresponds to small p-values (indicating closeness). The resulting histograms are shown in Figures 2 and 3 for the mouse and in Figures 4 and 5 for the human validation data. (Details of the validation data can be found in Additional file 1 for mouse and in Additional file 2 for human). We notice in Figures 2 and 4, which are based on the STRING functional network [19], that CellFateScout obtains the best score in 8 out of 11 cases for mouse and 6 out of 10 cases for human, respectively. However, employing iRefIndex [20] to construct the functional networks, we cannot find differences in result quality for any of the bioinformatics tools. Here, CellFateScout and ExprEssence are shown to be equally good, but only better to KeyPathwayMiner and jActiveModules by one or two cases. Also, randomization-based p-values do not show a significant difference for any tool in more than half of the cases, indicating a lower detection rate for small molecule effects in iRefIndex networks, as compared to STRING networks.

To study further the impact of the choice of the network construction source in our validation, we compared p-values for both sources and both species. Since samples are dependent and only the network construction methodology is varying, we used a one-sided paired t-test. For mouse, the mean of the differences of the p-values is −0.1938 between STRING and iRefIndex, and based on a one-sided paired t-test this difference is significantly smaller than 0 with p-value = 0.002. Thus, using STRING we observe an improvement of results; the active subnetworks/pathways are closer to the targets, and this is supported by smaller p-values. Based on the validation weighting function (see Methods), targets and genes directly connected to these targets obtain high weights and have a pivotal impact on the weighted mean distance. We noticed that on average, incoming links to these pivotal genes comprise 15.14% of all links in a STRING network, but only 8.56% in an iRefIndex network. In other words, given a more interconnected neighborhood close to the small molecule targets in STRING networks, more differentially expressed genes are connected to each other. Thus, in case of STRING, bioinformatics tools are able to report more links (being part of subnetworks or pathways) that connect genes in a neighborhood close to a small molecule target. For human, the mean of the differences of the p-values is -0.05209, which is not significantly smaller than 0 (one-side paired t-test, p-value = 0.187). Our findings confirm that in the context of our studies, STRING enables superior results than iRefIndex.

In summary, we find that CellFateScout together with STRING has the most success to discover pathways that are close to a small molecule’s known targets. Second best tool is ExprEssence, which is better than KeyPathwayMiner and jActiveModules by one or two cases. Our validation cannot confirm results from [9], where KeyPathwayMiner outcompeted jActiveModules. Limma, the gene-wise approach for identifying differentially expressed genes, has a best score in only one case study. This indicates that genes exhibiting highest change in expression do not have to be close to the targets of the small molecule under consideration. We also noticed that KeyPathwayMiner in 11 out of 20 cases in mouse and 9 out of 19 cases in human returns as output large subnetworks comprising of more than 20% of the genes from the analyzed network (see Additional file 1). Even when the output includes targets or genes close to targets, this indicates high recall at the expense of low precision, which can hamper clear and straightforward interpretation. In summary, our systematic validation suggests that CellFateScout is highly useful for active signaling pathway identification.

### Small molecule mechanisms database

Using CellFateScout together with expression data taken from the Connectivity Map project, small molecule targets from STITCH and functional networks from STRING, we performed a systematic search for active pathways triggered by small molecules and stored them in a Small Molecule Mechanisms Database as illustrated in Figure 1B. In turn, as shown in Figure 1C, this database can be queried, given a list of active signaling pathways derived from differential gene expression data, returning a set of small molecules that may trigger the same active pathways. A detailed description of the input, the output, the usage of our tool and of our methodology can be found in ‘Methods’.

More specifically, we developed a Small Molecule Mechanisms Database (SMMD) comprising 818 small molecule records (see below) associated with the mechanisms triggered by them. These mechanisms, presented as active signaling pathways, can, e.g., be used for scouting cell fate by small molecules that have specific desired effects as found by our plugin. Right now the database features only human mechanisms, however this can be extended to other species.

To construct the SMMD, we used the Connectivity Map (CMap, [16]) database, a collection of microarrays, where drug effects in human cells are measured in a large-scale, systematic way, focusing on expression profiles of the response of cell lines to 1309 small molecules. Specifically, the small molecules were investigated in various doses in 4 different cell lines (breast epithelial adenocarcinoma MCF7, prostate adenocarcinoma epithelial PC3, nonepithelial promyelocytic leukemia HL60 and malignant melanoma SKMEL5) by hybridization on 3 Affymetrix microarray platforms (HG-U133A, HT_HG-U133A, HT_HG-U133A_EA). Here, a record is a unique combination of a small molecule, its concentration, the cell line and the platform used. We ignore all records for which there are no microarray replicates in CMap (for case and control) or no targets in STITCH [21] connected to the small molecule with confidence of at least 0.9. All SMMD records are documented in Additional file 3. Microarray data is processed for every platform separately. We normalized all raw data from CMap (over 7000 arrays) with the aroma.affymetrix [22] R package for analyzing large Affymetrix data sets. In the CMap project, data were collected in multiple batches, each defined as a set of cases and controls performed together. For some of the database records, microarrays are taken from more than one batch. In order to remove batch-to-batch technical variation we follow [23] and apply ComBat [24]. Then, every SMMD record is analyzed individually. We construct networks of the top 2000 differentially expressed genes selected by limma. In agreement with conclusions from our validation effort, we employ STRING (but not iRefIndex) for network construction. In 73.58% cases, the known targets of a small molecule are not included in the network based on differential expression. Therefore, to improve the comprehensiveness of the database, we added curated knowledge about targets of small molecules from STITCH. More specifically, we added the known small molecule targets to the network together with neighborhoods of size large enough to connect these targets to the rest of the network. Finally, we run CellFateScout over all pathways (chains) in the network starting with a small molecule target gene, and for each small molecule, the 100 most active pathways along with their p-values are stored in the SMMD. The database is constructed with ObjectDB [25], a Java oriented database management system, and stored in one file. The user can view the SMMD file content with the ObjectDB Explorer tool, which is part of the ObjectDB project. The SMMD is available as Additional file 4 and also at https://sourceforge.net/projects/cellfatescout/.

Using CellFateScout, we can discover active signaling pathways. Having a list of these, we can perform an automated query in the Small Molecule Mechanisms Database to identify records of small molecules that may trigger the same pathway activity. For each active pathway, we thus search for SMMD records featuring database pathways with a low p-value and with matching genes from the active pathway. For an active pathway (AP) and a database pathway (DP) pair, we define a similarity score:

$$\mathrm{score}\left(\mathrm{AP},\mathrm{DP}\right)=\frac{\mathrm{overlap}\left(\mathrm{AP},\mathrm{DP}\right)}{\mathrm{pvalu}e_{\mathrm{AP}}+\mathrm{pvalu}e_{\mathrm{DP}}},$$

where the overlap in the numerator is defined as follows,

$$\mathrm{overlap}\left(\mathrm{AP},\mathrm{DP}\right)=\frac{\mathrm{number}\;\mathrm{of}\;\mathrm{genes}\;\mathrm{in}\;\mathrm{AP}\;\mathrm{and}\;\mathrm{in}\;\mathrm{DP}}{\mathrm{number}\;\mathrm{of}\;\mathrm{genes}\;\mathrm{in}\;\mathrm{AP}}.$$

We sort the query result based on the similarity score. Then, we collect the 100 best small molecule records with their respective database pathways and present these in CellFateScout.

### CellFateScout assumptions, application example, strengths and weaknesses

In contrast to bioinformatics tools designed to focus on finding pathways or connected subnetworks composed of genes that are significantly differentially expressed, CellFateScout uses the Latent Variable Model and considers the joint expression level of all genes in a pathway under investigation and thus takes into account the expression level of all genes in the entire network, and their interactions. Therefore, genes that have only few connections and that are not exhibiting significant expression change may still be considered components of active pathways. In such cases, signaling is done through e.g. phosphorylation of the protein product. This is an important aspect to consider when analyzing small molecule responses. Accordingly, there is evidence in drug target studies that significantly differential expression analysis is only partially effective in detecting target genes [26] and only a small fraction of targets are subject to significantly differential expression upon drug treatment [27]. Also, drug targets have more interactions with other proteins on average, but fewer than essential proteins [28], indicating that they are neither essential nor peripheral. Taking it all together, as in [29], we do not assume that drug targets are necessarily exhibiting significant change in expression; they are, however, located close to active signaling pathways and they initiate a cascade of signaling events that ultimately generates the observed gene expression response, as in [30].

**Figure 6:**
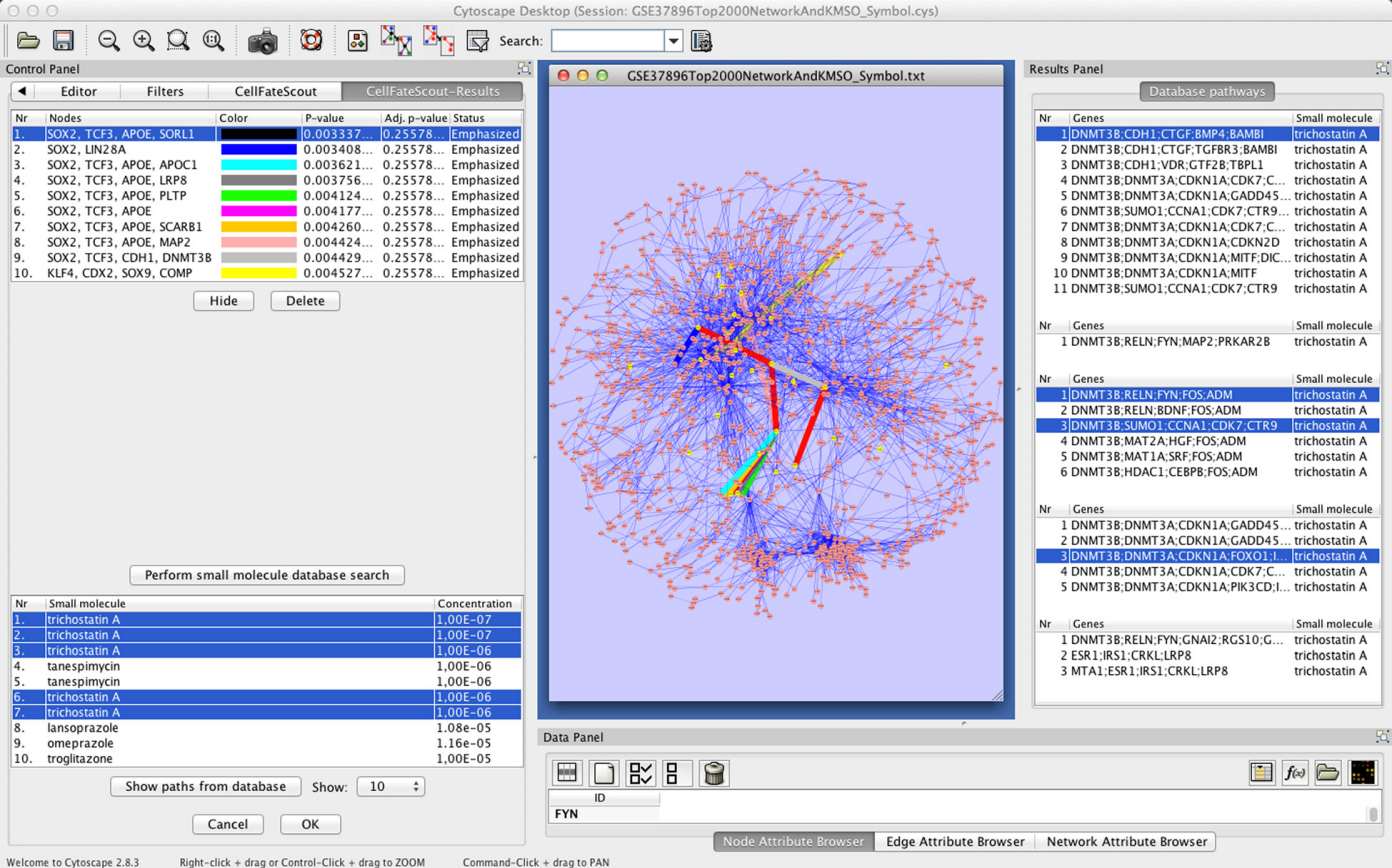
**Screenshot of CellFateScout result.** Active pathways are listed in the result table, top-left, and are given distinct colors and thicker edges to distinguish them in the Cytoscape network view, which is rendering the network visualization in the middle. Selecting an active pathway from the result table will select all its elements in the Cytoscape network view. There are two buttons below the result table, either to undo coloring of a specific pathway in the network view or to delete a pathway from the result table. Below the list of active pathways, the SMMD query result is shown in the bottom-left table, for up to 100 unique records. User selection of specific records will highlight all their pathways nodes and edges in the network view. The user can also highlight these pathways in the Cytoscape Result Panel (right), and there the user can investigate the pathways belonging to a small molecule record in detail. These pathways can also be highlighted in the network view.

Based on our findings, we constructed a Small Molecule Mechanisms Database. Rather than focusing on single genes as small molecule targets, we believe it is more useful and gives more insight to consider groups of genes that constitute active signaling pathways. Our database is a collection of small molecules and their target mechanisms in the form of active signaling pathways. Thus, we can utilize the SMMD to search for small molecules that have an expression signature closely matching the signature found in an experimental setup. An example of such a search and its result is given in Figure 6.

There are some limitations to our plugin. The most important caveat is a consequence of the noisiness and context-sensitivity of biological data, resulting in false positive results due to spurious expression values or network information. In particular, the activity of subnetworks, pathways or single interactions is a function of many confounding influences, ranging from obvious issues such as cell-line specific effects to more hidden ones, such as the temperature at which specific experiments were conducted. Moreover, high throughput technology is not all-encompassing and it may not detect all changes in the system under investigation. Limited accuracy and adequacy of the network structure in general is another caveat and limitation of network analysis; certain elements of biological systems are not readily portrayed in a network context [7]. Finally, as discussed above, gene expression may not necessarily reflect protein abundance.

More specifically to our method, in the Latent Variable framework, the network structure is represented as an adjacency matrix and its dimension increases with the number of genes included. Thus, calculations performed to estimate model parameters may be computationally demanding in case of large networks. Along these lines, CellFateScout gives the user a choice to focus only on nodes (genes) that she would like to investigate as start or end points of pathways. Performing exhaustive exploration of the pathways from all genes to all other genes may take a long time.

## Conclusions

CellFateScout is a powerful tool, easily usable as a plugin for Cytoscape, developed to support the design of experiments involving small molecules, with applications in cell transition research and drug repositioning. Having high throughput data and a functional network, the user can reveal active signaling pathways in the underlying network. These active signaling pathways may be prime candidates for more detailed modeling as in [31], to further our understanding of small molecule perturbation experiments. We evaluated CellFateScout on microarray gene expression datasets with small molecules involved, and compared results with other bioinformatics tools. Validation showed that our plugin predicts more precisely the small molecule effects. CellFateScout evaluates pathways with the Latent Variable framework that considers the joint expression of a whole pathway, whereas most of the network-based bioinformatics tools search for subnetworks centered around significantly differentially expressed genes. Since the direct targets of small molecules often do not exhibit high expression change, methods based on differentially expressed genes may not find genes close to target genes. Chromatin modification drugs such as valproic acid (see Methods) considered in our validation may be examples for this phenomenon. They may modify chromatin by inhibition of histone deacetylases without significant change of histone deacetylase transcription, thus their target genes do not necessarily occur in microarray studies as differentially expressed genes or at the top of a list of these. Our validation in human and mouse also showed that genes with the highest expression change found with limma are not usually close to direct targets of small molecules.

## Methods

The CellFateScout tool is designed as a Cytoscape 2.8.3 [32] plugin. Cytoscape is a software for complex network analysis and visualization implemented in Java, which can integrate additional data like microarray gene expression sets, and be extended by third party plugins. The underlying methodology of CellFateScout is an adaptation of the “Latent Variable” approach [13,14] to pathway activity estimation.

### Pathway evaluation – the statistical model

We define a functional network as a gene/protein interaction network. Thus, the nodes in such a network represent genes and gene products (proteins), and the links between the nodes may be regulatory (where a protein controls the expression of a gene) or physical (where two proteins interact). As in other work along the same lines, gene expression data is used as a proxy for protein expression (which is hard to obtain in a quantitative manner), and it is assumed that gene and protein expression correlate. To simplify terminology, we therefore refer to ‘genes’. In this study, a pathway is a linear chain in a functional network. Such a linear chain may reflect the backbone of a canonical pathway such as a KEGG [33] pathway, but it may also reflect pathway crosstalk. Per default, we investigate all linear chains in a functional network, so we consider all pathway-related information encoded by the network.

We define the *activity* of a pathway as the amount of change in expression level across its nodes (genes), which can be thought of as the information (or signal) flow along the pathway. Pathway activity is discovered depending on a specific formula that varies depending on the aims of the study. Usually, change in expression level is considered for a pair or a set of linked genes, following the idea that signaling includes patterns of gene activity that are measurable by differential expression data along the links between the genes. More specifically, this measurement will be done in our case by adapting the “Latent Variable” approach, as described in the next paragraph. We define the *active signaling pathways* in a network as a set of pathways with an activity above a specific threshold.

The Latent Variable Model [13,14] can exploit information from an input functional network combined with high-throughput expression data. This approach incorporates directly the underlying network structure into a model with both directed and undirected edges. It assumes that the expression level of each node (gene) is based on its own expression and on the influence of the expression of other genes. The contribution of a gene *g* to its expression level is called a latent variable, and is modeled as a normally distributed random variable. On the other hand, the influence of other genes on the expression level of *g* is calculated from the expression level of the genes linked to *g,* considering the incoming edge weights. The Latent Variable Model can be represented as a Mixed Linear Model. Then, the fixed effects of the latent variables can be tested. In our adaptation, we consider pathways in the form of linear chains. The authors of [14] then consider a statistical test for each specific subnetwork, where the null hypothesis is assuming that there is no change in time of a gene’s self-contribution to its expression level and there is no change of its influence to other genes in this subnetwork. The testing is done using the Wald test statistic that under the null hypothesis follows approximately a t-distribution. Using this framework, [14] developed a general inference procedure that can be used to identify active subnetworks in complex experiments over time. This framework requires extensive adjustment of the statistical analysis for every new application [34]. In our case, the adjustment was done as follows. To be simple and applicable to experiments involving two conditions, e.g. before and after an intervention, or case and control, we simplified the Latent Variable Model framework for two conditions and an underlying network, the structure of which does not change over time. In general, this simplified framework allows researchers to investigate the activity of any kind of subnetwork of interest, but the scope of CellFateScout is the identification of active pathways in the form of a linear chain, simplifying and speeding up the analyses. In particular, with our adaptation of the method we determine significance estimates (p-values) for the activation status of all pathway chains that we choose to explore, rank them based on the p-value and highlight them in the network. The same approach is also utilized for the small molecule database construction.

### Validation methodology

We selected datasets of small molecules with corresponding gene expression and functional network data and developed a validation scheme as described below that estimates the closeness between known targets of the small molecules and the active subnetworks/pathways (or corresponding gene sets) found by the bioinformatics tools.

For our validation effort, we collected publicly available microarray experiments for two organisms, mouse and human, where only one small molecule is investigated per microarray. Target genes of these small molecules had to be included in the STITCH database version 3.1, a chemical-protein interactions resource [21]. There, every interaction between a small molecule and a gene/protein has a confidence score of at most 1. In order to consider only small molecules with a high likelihood that there exist correctly curated target genes, we used a confidence threshold of 0.9. Finally, we required microarray data to meet the criterion of having replicates for case and for control.

For mouse, we investigated small molecules that are known to modulate embryonic stem cell fate and somatic cell reprogramming. Zhang collected these in a review [35]; out of the 39 listed there, 12 have high confidence targets in STITCH and feature experiments with replicates in the Gene Expression Omnibus (GEO) repository as of January 2013 [36]. From this set of small molecules we do not consider retinoic acid due to its high number of 84 target genes, and we therefore investigated 11 small molecules. A summary of our data is presented in Table 1. We imported normalized microarray expression sets from the GEO database into the R statistical computing and graphics environment [37] using the Bioconductor package GEOquery [38]. These expression sets can be directly passed on to Cytoscape and used with CellFateScout. Other tools required additional steps in data preparation. For jActiveModules, we had to assign a p-value from differential expression analysis to the genes and for this we used the R limma package. KeyPathwayMiner requires a selection of differentially expressed genes from the network. Here, we followed the original manuscript and processed the data as described there.

**Table 1:** Mouse data used for validation

**Mouse**						
**Small molecule**	**Number of targets in STITCH**	**GSM sample number**		**Publication**	**STRING Network (nodes/edges/targets in whole STRING network)**	**iRefIndex Network (nodes/edges/targets in whole iRefIndex network)**
		**Perturbation (case)**	**Reference (control)**			
Ascorbic Acid	6	854784, 854785, 854786, 854787	854788, 854789, 854790, 854791	[39]	189/162/2	128/97/1
Dorsomorphin	6	677043, 677044	677041, 677042	[40]	258/202/6	161/131/5
Fasudil	1	634510, 634518, 634526, 634534	634509, 634517, 634525, 634533	[41]	338/445/1	246/223/1
Rosiglitazone	10	794249, 794250, 794251	794242, 794243, 794244, 794245	[42]	376/504/10	233/220/6
SAHA	1	890615, 890616, 890617	890678, 890679, 890680, 890681, 890682, 890683, 890684, 890685, 890686, 890687, 890688, 890689, 890690, 890691	[43]	377/389/1	220/197/1
SB203580	20	37115, 37116	37106, 37107	[44]	571/781/19	337/30717
SB431542	1	571146, 571147, 571148, 571149	571150, 571151, 571152, 571153	[45]	372/415/1	-
SU5402	2	400317, 400318, 400319	400305, 400306, 400307	[46]	412/530/2	229/203/1
Trichostatin A	12	8898, 8902	8900, 8904	[47]	564/1044/9	423/484/11
U0126	13	377044, 377045, 377046	376950, 376951, 376952	[48]	278/263/13	120/79/11
Valproic Acid	19	234805, 234806, 234807, 234808	234794, 234795, 234796, 234797	[49]	332/614/17	-

For human, we selected small molecules applied in chromatin-modification therapeutic studies in cancer and a summary of such studies (clinical trials) is assembled in Mai [50] in the supplement therein. We applied the same criteria as for mouse to narrow down our set of small molecules, filtering drugs with known curated target genes in STITCH. Then, we intersected them with all small molecules that were used in the Connectivity Map. This intersection is meaningful because both the clinical trials of Mai and the Connectivity Map data are based on cancer cell lines. We further focused only on microarrays that were performed with the breast cancer epithelial cell line MCF7. Finally, from the 14 drugs surviving all filters described, we omitted those with a high number of more than 100 human target genes. For the resulting 10 small molecules, the corresponding microarrays and their controls were normalized (see SMMD construction, above), processed as for mouse (see above) and passed for validation as described below. A summary of the human data can be found in Table 2.

**Table 2:** Human data used for validation

**Human**					
**Small molecule**	**Number of targets in STITCH**	**Concentration**	**Array**	**STRING Network (nodes/edges/targets in whole STRING network)**	**iRefIndex Network (nodes/edges/targets in whole iRefIndex network)**
Azacitidine	8	0.0000164	HT_HG-U133A	1539/4029/8	1559/5804/7
Doxorubicin	48	0.0000068	HT_HG-U133A	1504/3937/44	1574/5896/45
Etoposide	41	0.0000068	HT_HG-U133A	1510/4171/37	1540/5514/39
Hydralazine	3	0.0000204	HT_HG-U133A	1468/2977/3	1372/3633/3
Lithocholic Acid	59	0.0000106	HT_HG-U133A	1414/2745/59	1282/2649/54
Methylprednisolone	6	0.0000106	HT_HG-U133A	1411/3239/5	1389/3653/5
Prednisone	1	0.0000112	HT_HG-U133A	1433/2988/1	-
Tamoxifen	56	0.000007	HT_HG-U133A	1468/3400/56	1505/4969/49
Valproic Acid	20	0.001	HT_HG-U133A	1386/2870/19	1451/4382/17
Vorinostat	22	0.00001	HT_HG-U133A	1505/4302/22	1580/6135/22

Focusing on protein-protein and protein-gene interactions to identify active signaling pathways from expression data (see also [8,26,51-54]), we used two distinct database repositories to construct functional networks for both species, mouse and human, setting the appropriate species as a database retrieval parameter. In a first approach, we utilized information from the STRING database (version 9.0) [55]. Their website allows to import and construct protein/gene interaction and regulation networks for up to 2000 genes, and thus, for each small molecule/microarray experiment, we based our network construction upon the top 2000 differentially expressed genes as calculated by GEO2R, a GEO tool that in turn employs the limma R package. We then set a high confidence STRING score threshold of 0.9 for filtering out unreliable links and deleted from the network all genes that are not linked to any other gene. In a second approach, we considered the same top 2000 genes, but we used functional network information taken from the iRefIndex database version 9.0 [20]. We considered networks from both repositories as undirected. Summaries of our data assembly and network constructions are also given in Tables 1 and 2. (For two small molecules in the mouse data, SB431542 and Valproic Acid, and for Prednisone in the human data, target genes are not found in the complete mouse or human iRefIndex network, thus it is not possible to conduct the validation for these small molecules in case of iRefIndex).

We defined the *neighborhood* of a small molecule within a network as its target genes and their neighbors, up to a specific distance. We expected the genes in the neighborhood to be affected by the small molecule, that is, to feature a change in their expression. Our validation is thus based on the question: are the active pathways we find close to the known targets? In other words, in how far does the neighborhood of the small molecule, based on its targets, coincide with the pathways it is supposed to activate, based on the expression data of the experiment measuring its effects?

All of the bioinformatics tools we compare represented different ways to identify such small molecule effects, by gene-wise independent testing, highlighting links or identifying active subnetworks. We used these tools together with expression experiments and corresponding networks to compare their accuracy in identifying relevant pathway activity patterns, as follows. We ran our plugin on all datasets we collected (Tables 1 and 2) and performed an exhaustive search investigating every pathway of size up to 6, consisting of 7 genes connected by 6 links; 6 is the first integer greater than the right-hand side of the 95% confidence interval for the average path length of all constructed networks, that is (4.42, 5.22). Each exhaustive search resulted in a listing of the 10 most active pathways, based on the gene expression data of the microarray and the corresponding network. Then, we ran other tools with default settings on the same datasets and collected the results top-down (best results first) to obtain (almost) the same number of genes as we did using CellFateScout. To facilitate comparison of the gene lists from limma, links (gene pairs) from ExprEssence, active pathways from CellFateScout, and active subnetworks from jActiveModules and KeyPathwayMiner, we treated all results as sets of individual genes embedded in a network. In order to analyze the proximity of these gene sets to the small molecule targets as defined by STITCH, we defined a formula for the distance between a gene and a small molecule in terms of a network, and, more generally, between a set of genes and a small molecule (see below). Small molecule target genes and their neighborhoods often do not occur in the network constructed out of the 2000 most differentially expressed genes, since signaling is not always reflected by transcriptional change, but also e.g. by phosphorylation of the protein product. For this reason we calculated the distance of a gene to a small molecule considering the whole network of interactions from STRING (with confidence score at least 0.9) or from iRefIndex. The distances between the targets and the genes found by the bioinformatics tools can be found in Additional file 1 for mouse and Additional file 2 for human.

The distance of a single gene to a small molecule is the shortest distance of this gene to its closest small molecule target in the network. Further, we introduced a weighting function so that a weight is assigned to each distance based on the size of the neighborhood of the small molecule. This weight will be high if the gene is near a small molecule and the number of genes of the same, or smaller, distance to this molecule is low. With this weighting we give strong weight to the distances of genes proximal or coinciding with target genes. The weight of distance *i* is

$$\mathrm{weight}\left(i\right)=\sqrt[{3}]{1-\frac{n_{i}-1}{N}}.$$

**Figure 7:**
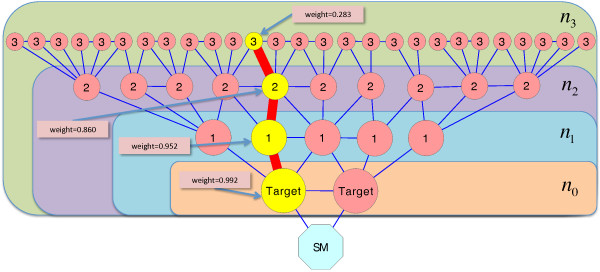
**Visualization of the weighting function used in our validation approach.** Using the weighting function promotes gene sets proximal or coinciding with target genes. A weight is assigned to a gene based on its distance to the small molecules and the size of the neighborhood of the small molecule. In this toy example, we have network nodes of distance 0 (targets), 1, 2 and 3 to the small molecules targets and *n*_*i*_ represents the number of genes of distance *i* or less to these targets. The weight of a pathway gene (colored in yellow) is calculated based on the weighting function and e.g. for a node of distance 2 in this toy example, the weight is 0.860.

Here, *n*_*i*_ indicates the number of genes in the network of distance *i* or less (with respect to the gene in question) and *N* the number of all nodes in the network. Thus, the weighting function is monotonically decreasing with respect to *i*, when *N* is fixed. A visualization of this approach is presented in Figure 7. Next, the distance of a set of genes to a small molecule is defined as the weighted mean of the individual distances. This gives a score for the distances of a set of genes and compensates that some bioinformatics tools return a variable number of genes; with more genes in a gene set, the expected number of hits (genes near targets) is also increasing. Finally, we challenged all bioinformatics tools against results from randomization. Thus, we applied a bootstrapping technique to create a sample distribution by generating 10000 random gene sets of the same size as the average of the resulting pathways and calculating the distance scores of these random sets to the small molecule under investigation. Using this random sample we calculated one-sided p-values. These p-values are easier to interpret, more meaningful and easier to compare than weighted mean distances: They describe how much better the results taken from the bioinformatics tools are, compared to “pathways” based on selecting genes randomly, and they are not affected by topological bias in the network.

As a result of our validation, we obtained p-values, measuring our improvement over chance findings, i.e. how much closer to the known targets are the active pathways we find, compared to random pathways, and how significant is this difference? We obtained the same kind of p-values for the other bioinformatics tools. In order to visualize the validation results, we transformed the resulting p-values from the randomization test, taking the − *log*_10_(*p* − *value*).

### CellFateScout input

The plugin requires a functional network and at least two high-throughput datasets (differentially describing two conditions) imported into Cytoscape. The network consists of nodes and edges. As described, with respect to the nodes, we do not distinguish between genes and proteins. Thus, the edges may describe physical interactions between gene products (proteins) as well as regulatory interactions between genes and proteins. Both kinds of interactions are called *links*. The former are usually undirected, while the latter may be directed (stimulations, inhibitions). Links may be weighted by their strength, which may be measured experimentally. By default, the network is treated as undirected and unweighted. Nevertheless, the user can select edges and define their directionality and assign weights. The expression values must be imported as node attributes and due to the statistical model utilized, only data with at least two replicates per condition can be processed.

### Interface of the CellFateScout plugin and output

The CellFateScout user interface is implemented in a way that it is easy to grasp for non-computer scientists, but still functional and comprehensive. A tutorial is available at http://sourceforge.net/projects/cellfatescout and as Additional file 5. Before the user starts the plugin, a Cytoscape session with a valid network and expression data must be uploaded. In a first step, the user may explicitly define edge directionalities. When there is additional information about the links, CellFateScout also allows assigning weights to these. Thus, the user can control how pathways will be investigated and which links have high priority.

Complex networks feature a high fraction of all possible links and analyzing all resulting pathways is very often neither possible nor necessary. Therefore, the plugin lets the user decide how to explore the network, by marking the nodes or edges that correspond to the start and to the end of the pathways to be explored. The user tags these nodes or edges in the Cytoscape network view. Additionally, CellFateScout allows selecting the entire neighborhood of a specified node. An edge is considered as the set of its two nodes. These multiple ways of node and edge selection give the user the opportunity to exploit the network in a way that suits best. In the minimalist scenario the user can select a single node for the start and for the end, whereas selecting all nodes as start as well as end points will trigger an exhaustive exploration. Finally, the user selects Cytoscape node attributes representing two conditions (e.g. case and control) from a high-throughput experiment. After computation, the most active signaling pathways with lowest p-values in the input Cytoscape network are shown in a table and marked by colors in the table and the Cytoscape network view. We also provide adjusted p-values after multiple testing correction using the Benjamini-Hochberg procedure [56]. The resulting pathways are sorted in the table from most to least significant (from low to high p-value), and delineated by color. If the resulting pathways have overlapping edges in the Cytoscape network view, colors are given based on rank. To keep the view simple and readable, by default we only consider the top ten pathways with top ranks. Selection of pathways in the result table will select all their elements, nodes and edges, in the Cytoscape network view. We also provide features that allow the user to delete pathways from the results table. When a pathway is deleted from the results table, the next significant pathway in the ranking that was not appearing in the table will be shown and displayed in the network view.

Once we know the activity pattern of the signaling pathways in the network, we can perform an automatic similarity search in the Small Molecule Mechanisms Database (SMMD) for matching pathways based on Connectivity Map data. Only user-selected active pathways from the results table will be queried for matching pathways. The matching pathways from the SMMD are associated with small molecules, each with information about the small molecule concentration, the cell line with which the experiment was conducted and the microarray platform used. (In each SMMD record, a small molecule and its associated information are connected to the pathways being activated). The matching database pathways are sorted based on a similarity score (see above) and up to 100 unique records with database pathways having the best similarity scores are shown. User selection of specific records will highlight, for all matching pathways, their nodes and edges in the Cytoscape network view. To investigate the pathways belonging to a specific record, the user can obtain their description in a Cytoscape Results Panel. In turn, the described pathways can be highlighted one-by-one in the Cytoscape network view by selecting them in the Results Panel. Since not all nodes or edges of a pathway belonging to a record have to be present in the original network for which the matching pathway was calculated, only some elements may be highlighted in the Cytoscape network view. An example view of a Cytoscape session while using CellFateScout is presented in Figure 6.

## Abbreviations

SMMD: Small molecule mechanism database; CMap: Connectivity map; GEO: Gene expression omnibus; SM: Small molecule; AP: Active pathway; DP: Database pathway.

## Competing interests

The authors declare that they have no competing interests.

## Authors’ contribution

MS developed the software, analyzed the data and wrote the paper. VL contributed to data analysis and paper writing. GF conceived the study and contributed to paper writing. All authors read and approved the final manuscript.

## Supplementary Material

Additional files 1 **Mouse validation summary.** Table that summarizes detailed results from mouse validation including weighted means distances, p-values, and the number of genes at a certain distance to the small molecules. Click here for file

Additional files 2 **Human validation summary.** Table that summarizes detailed results from human validation including weighted means distances, p-values, and the number of genes at a certain distance to the small molecules. Click here for file

Additional files 3 **Small Molecule Mechanisms Database records.** Summary of all small molecules from Connectivity Map included in the SMMD, including their respective concentration, cell line and Affymetrix microarray platform. Click here for file

Additional files 4 **Small Molecule Mechanisms Database.** The database is constructed with ObjectDB (http://www.objectdb.com), a Java oriented database management system. Content of this file can be viewed with the ObjectDB Explorer tool, which is a part of the ObjectDB project. This file is also available at http://sourceforge.net/projects/cellfatescout. Click here for file

Additional files 5 **CellFateScout step-by-step tutorial for a case study.** Tutorial that presents how CellFateScout can be utilized on an expression dataset. For this, we used the public microarray expression dataset available at GEO [36] under accession number GSE37896 [57]. This study is measuring gene expression change in iPS induction by lentiviral Yamanaka factors applied to adipose stem cells. Click here for file
