# Supplementary material for: CellFateScout – a bioinformatics tool for elucidating small molecule signaling pathways that drive cells in a specific direction
Source: Cell Commun Signal. 2013 Nov 8;11:85. doi: 10.1186/1478-811X-11-85 (PMC3833265; doi:10.1186/1478-811X-11-85)
Supplement: Additional files 5 — CellFateScout step-by-step tutorial for a case study. Tutorial that presents how CellFateScout can be utilized on an expression dataset. For this, we used the public microarray expression dataset available at GEO [36] under accession number GSE37896 [57]. This study is measuring gene expression change in iPS induction by lentiviral Yamanaka factors applied to adipose stem cells. [file 1478-811X-11-85-S5.ppt]

## Slide 1
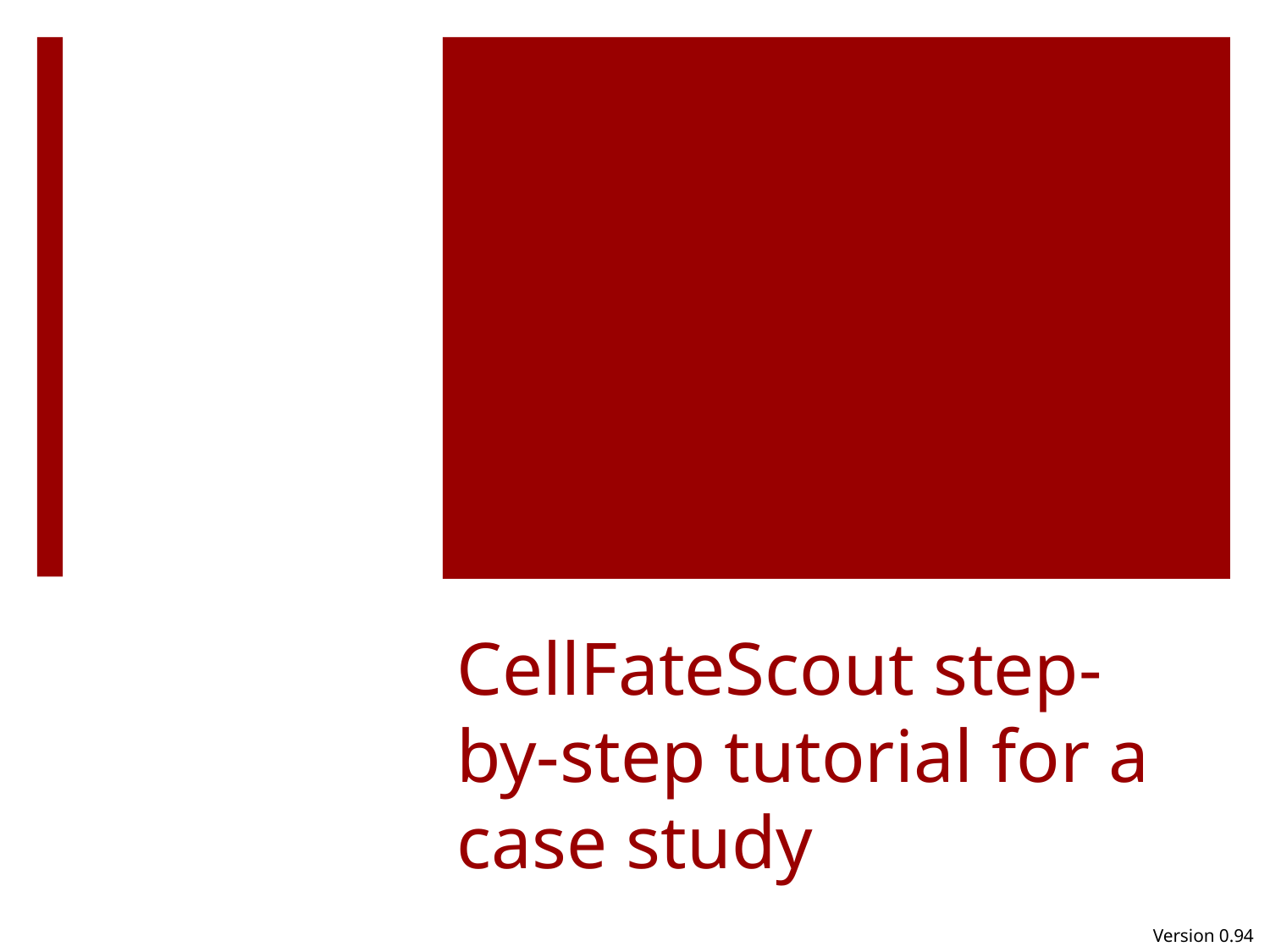

# CellFateScout step-by-step tutorial for a case study
Version 0.94

## Slide 2
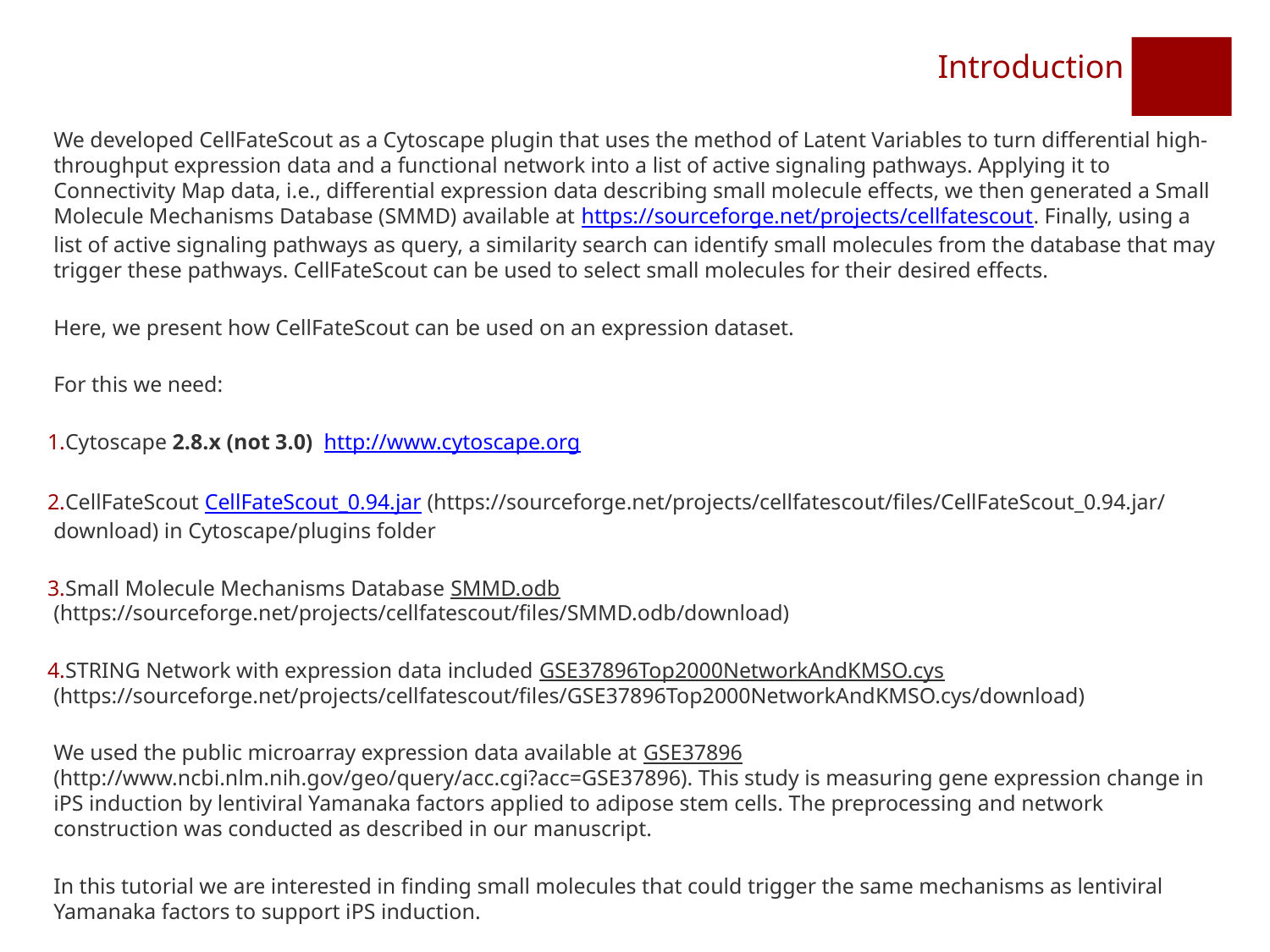

Introduction
We developed CellFateScout as a Cytoscape plugin that uses the method of Latent Variables to turn differential high-throughput expression data and a functional network into a list of active signaling pathways. Applying it to Connectivity Map data, i.e., differential expression data describing small molecule effects, we then generated a Small Molecule Mechanisms Database (SMMD) available at https://sourceforge.net/projects/cellfatescout. Finally, using a list of active signaling pathways as query, a similarity search can identify small molecules from the database that may trigger these pathways. CellFateScout can be used to select small molecules for their desired effects.
Here, we present how CellFateScout can be used on an expression dataset.
For this we need:
Cytoscape 2.8.x (not 3.0)  http://www.cytoscape.org
CellFateScout CellFateScout_0.94.jar (https://sourceforge.net/projects/cellfatescout/files/CellFateScout_0.94.jar/download) in Cytoscape/plugins folder
Small Molecule Mechanisms Database SMMD.odb (https://sourceforge.net/projects/cellfatescout/files/SMMD.odb/download)
STRING Network with expression data included GSE37896Top2000NetworkAndKMSO.cys (https://sourceforge.net/projects/cellfatescout/files/GSE37896Top2000NetworkAndKMSO.cys/download)
We used the public microarray expression data available at GSE37896 (http://www.ncbi.nlm.nih.gov/geo/query/acc.cgi?acc=GSE37896). This study is measuring gene expression change in iPS induction by lentiviral Yamanaka factors applied to adipose stem cells. The preprocessing and network construction was conducted as described in our manuscript.
In this tutorial we are interested in finding small molecules that could trigger the same mechanisms as lentiviral Yamanaka factors to support iPS induction.

## Slide 3
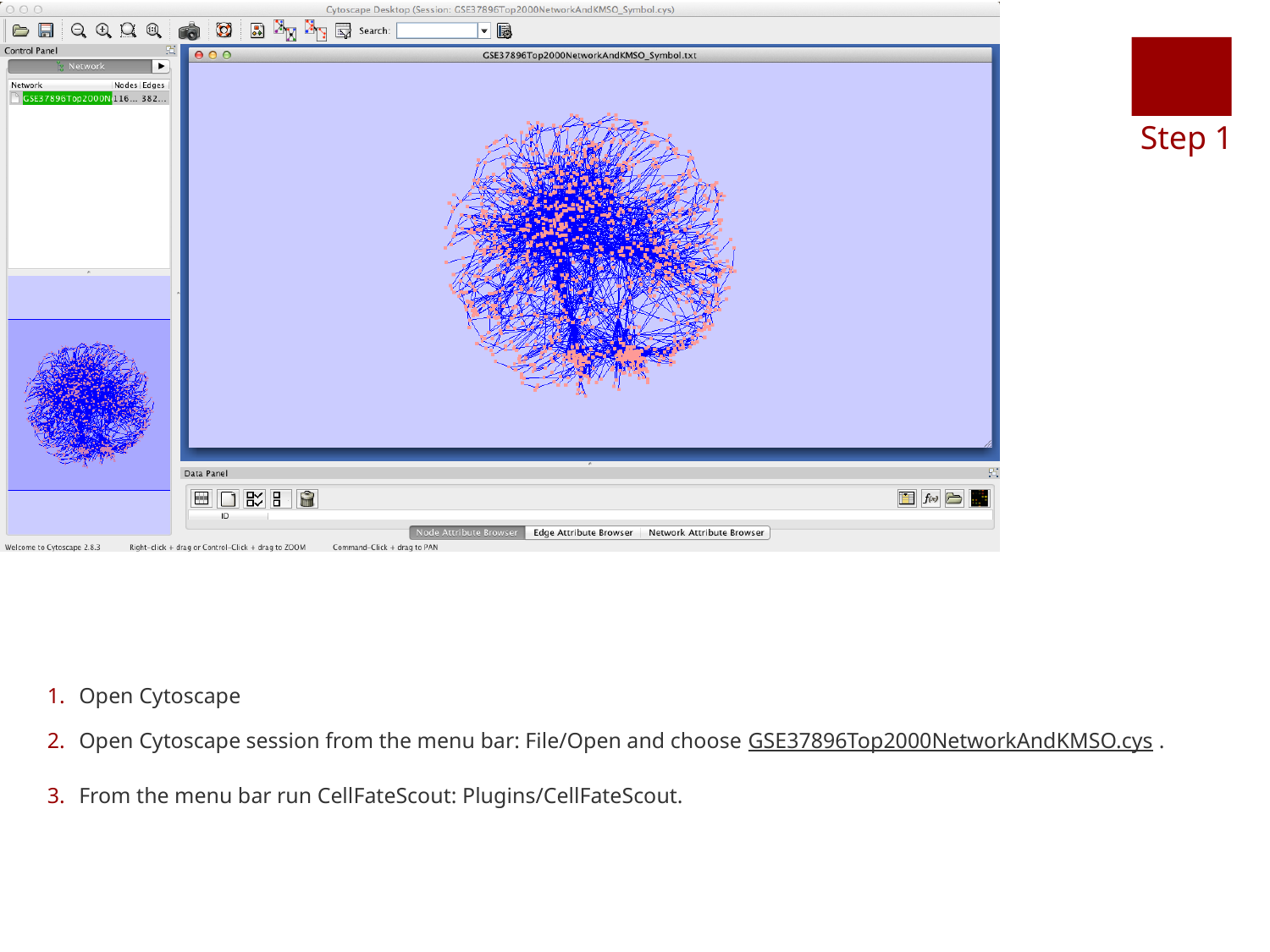

Step 1
Open Cytoscape
Open Cytoscape session from the menu bar: File/Open and choose GSE37896Top2000NetworkAndKMSO.cys .
From the menu bar run CellFateScout: Plugins/CellFateScout.

## Slide 4
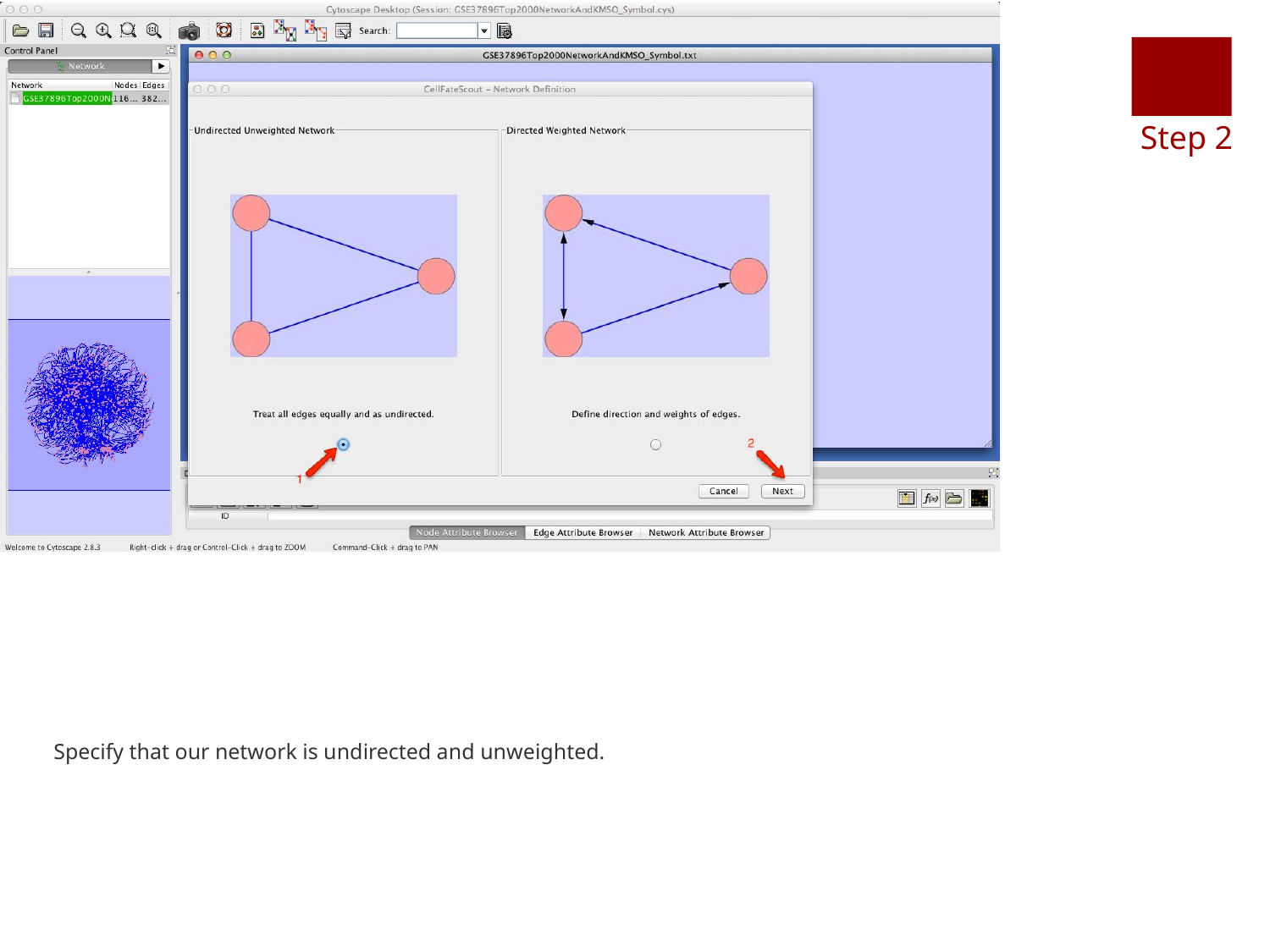

Step 2
Specify that our network is undirected and unweighted.

## Slide 5
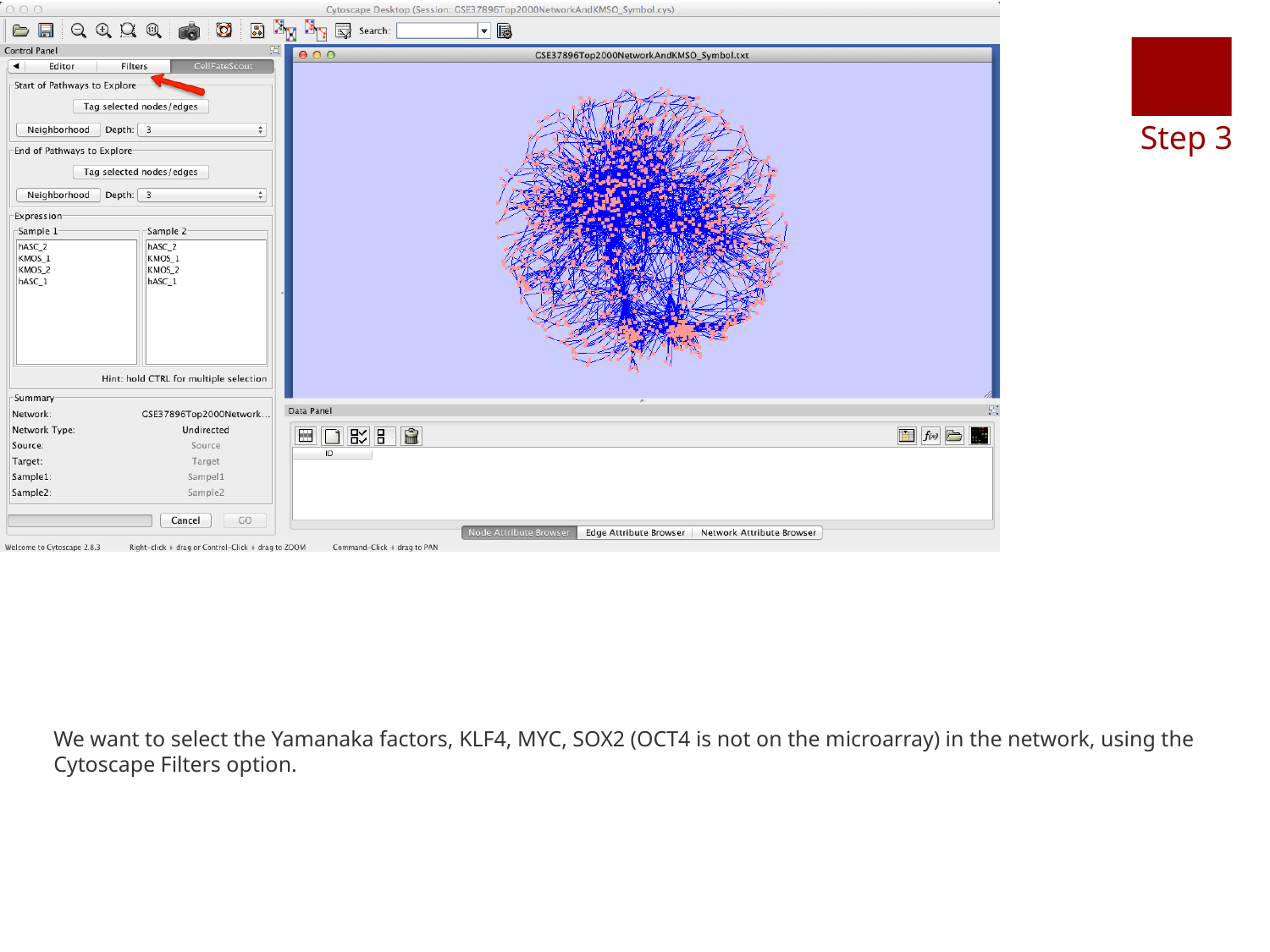

Step 3
We want to select the Yamanaka factors, KLF4, MYC, SOX2 (OCT4 is not on the microarray) in the network, using the Cytoscape Filters option.

## Slide 6
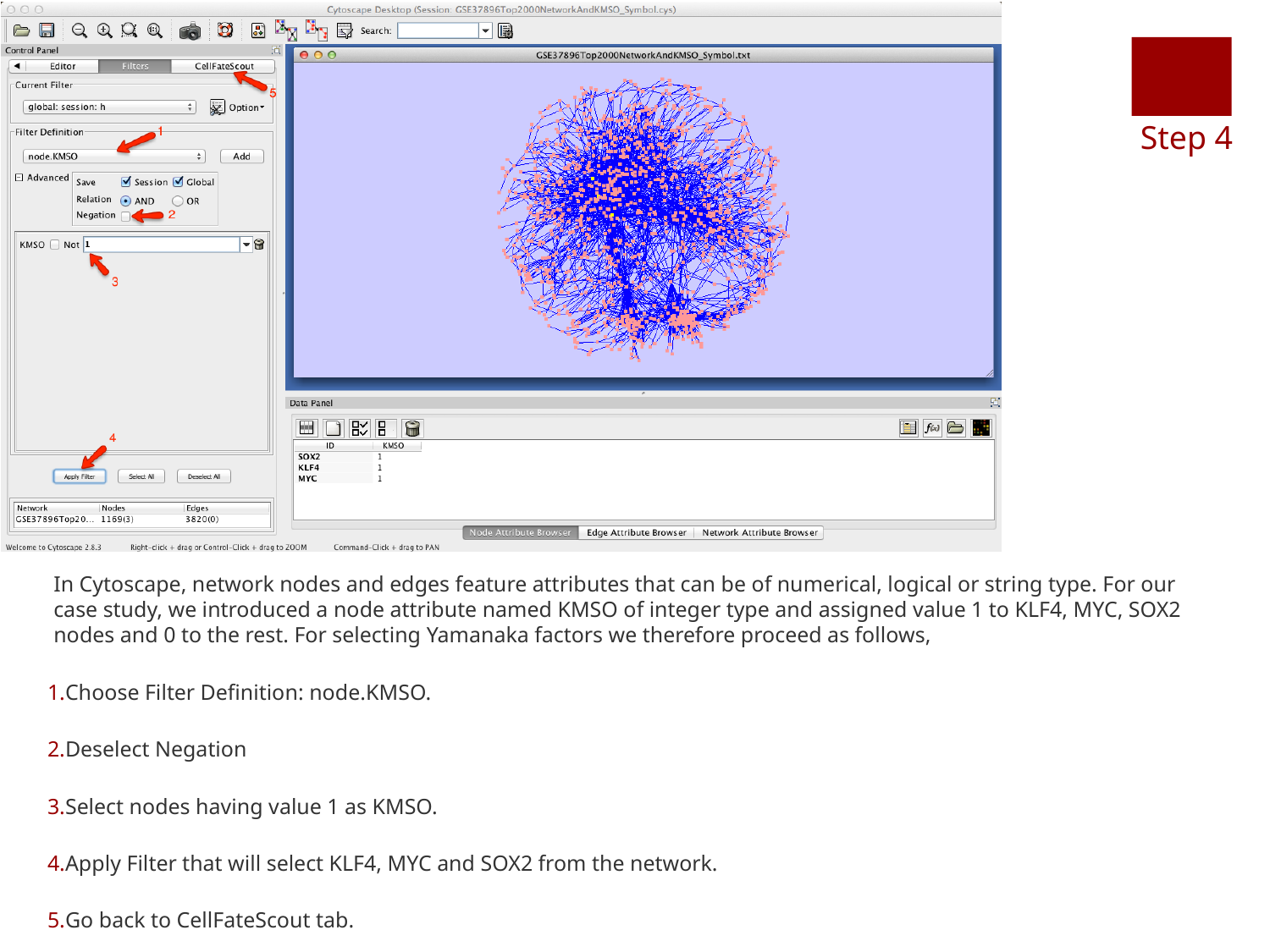

Step 4
In Cytoscape, network nodes and edges feature attributes that can be of numerical, logical or string type. For our case study, we introduced a node attribute named KMSO of integer type and assigned value 1 to KLF4, MYC, SOX2 nodes and 0 to the rest. For selecting Yamanaka factors we therefore proceed as follows,
Choose Filter Definition: node.KMSO.
Deselect Negation
Select nodes having value 1 as KMSO.
Apply Filter that will select KLF4, MYC and SOX2 from the network.
Go back to CellFateScout tab.

## Slide 7
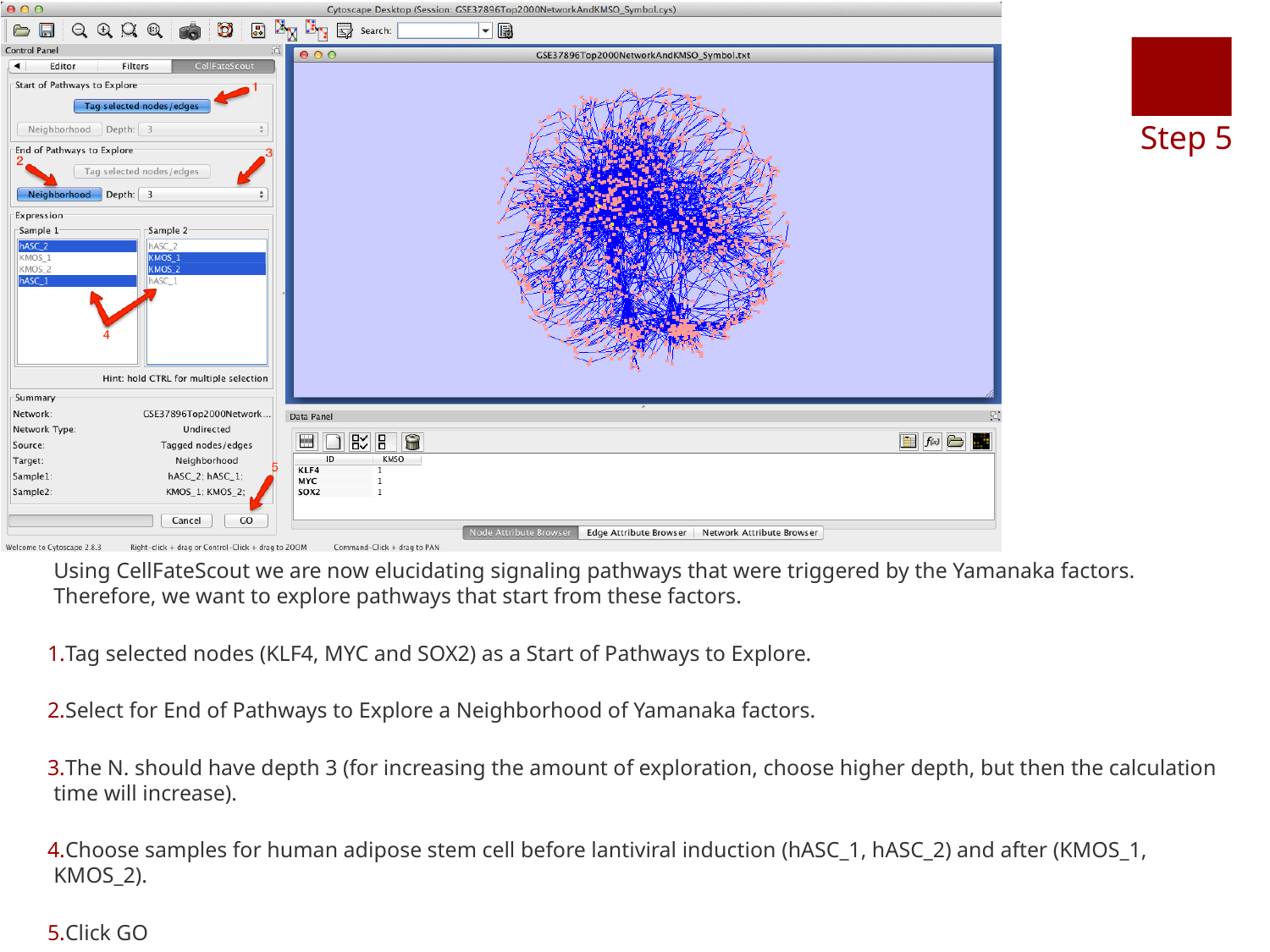

Step 5
Using CellFateScout we are now elucidating signaling pathways that were triggered by the Yamanaka factors. Therefore, we want to explore pathways that start from these factors.
Tag selected nodes (KLF4, MYC and SOX2) as a Start of Pathways to Explore.
Select for End of Pathways to Explore a Neighborhood of Yamanaka factors.
The N. should have depth 3 (for increasing the amount of exploration, choose higher depth, but then the calculation time will increase).
Choose samples for human adipose stem cell before lantiviral induction (hASC_1, hASC_2) and after (KMOS_1, KMOS_2).
Click GO

## Slide 8
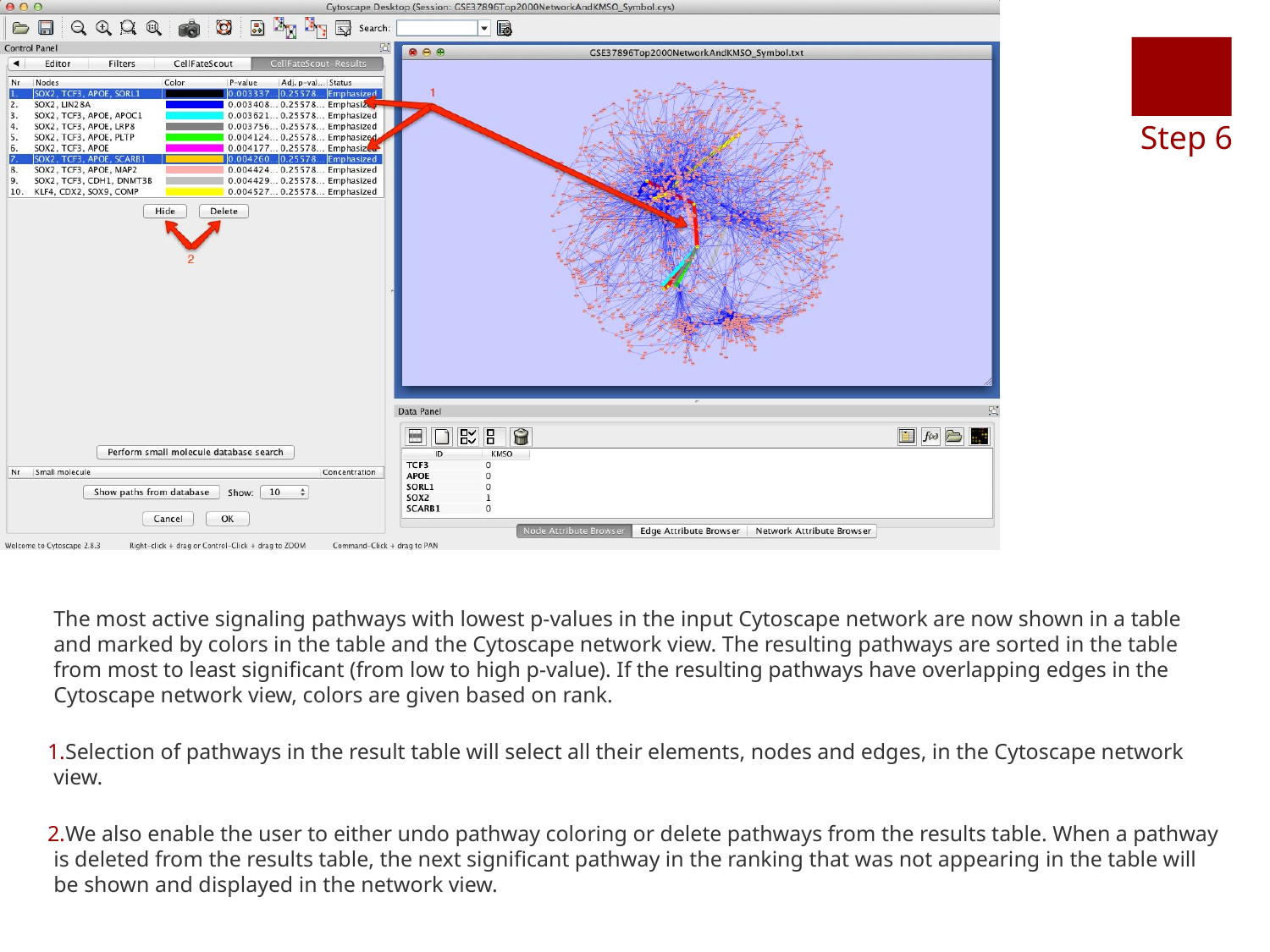

Step 6
The most active signaling pathways with lowest p-values in the input Cytoscape network are now shown in a table and marked by colors in the table and the Cytoscape network view. The resulting pathways are sorted in the table from most to least significant (from low to high p-value). If the resulting pathways have overlapping edges in the Cytoscape network view, colors are given based on rank.
Selection of pathways in the result table will select all their elements, nodes and edges, in the Cytoscape network view.
We also enable the user to either undo pathway coloring or delete pathways from the results table. When a pathway is deleted from the results table, the next significant pathway in the ranking that was not appearing in the table will be shown and displayed in the network view.

## Slide 9
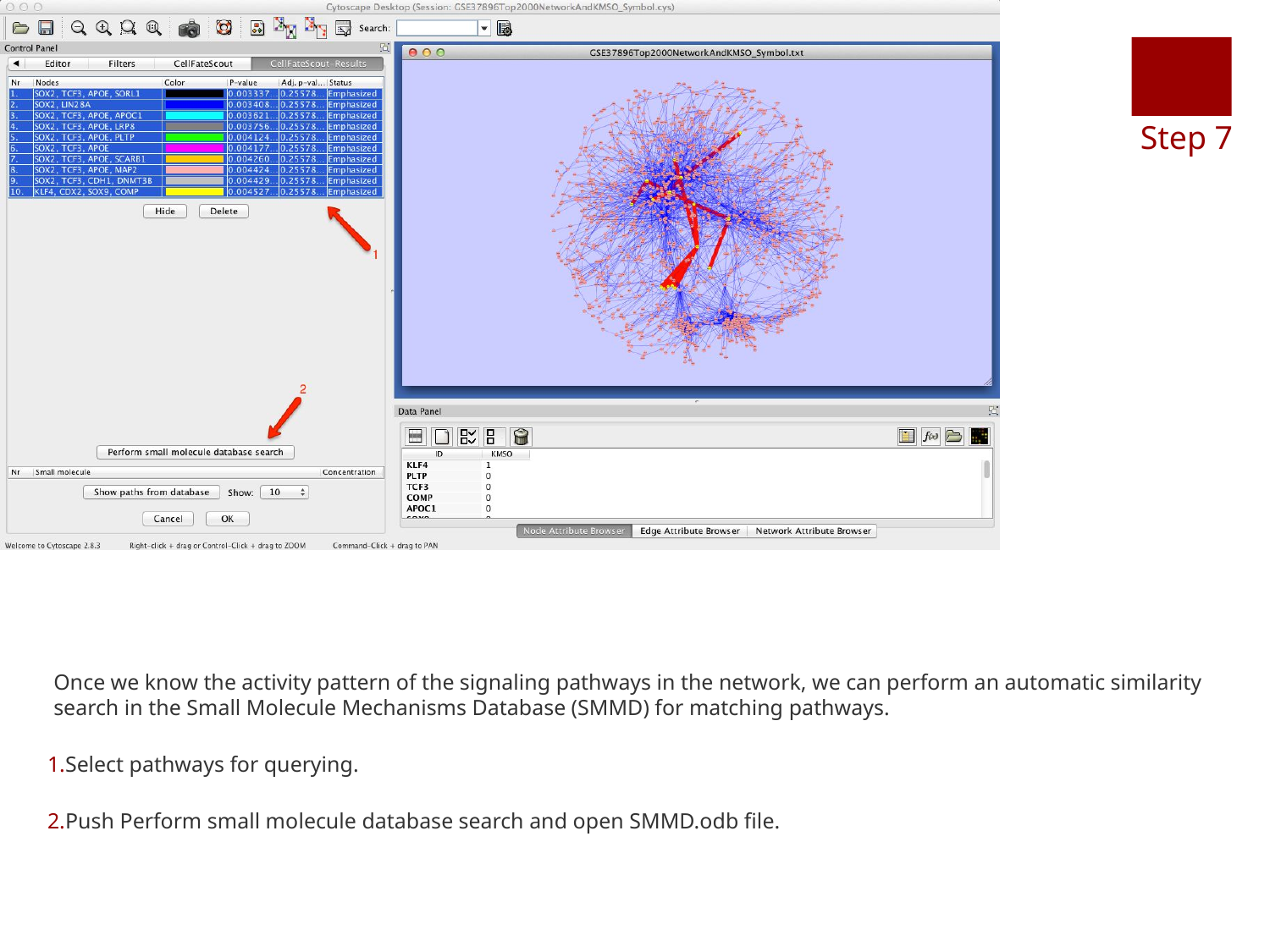

Step 7
Once we know the activity pattern of the signaling pathways in the network, we can perform an automatic similarity search in the Small Molecule Mechanisms Database (SMMD) for matching pathways.
Select pathways for querying.
Push Perform small molecule database search and open SMMD.odb file.

## Slide 10
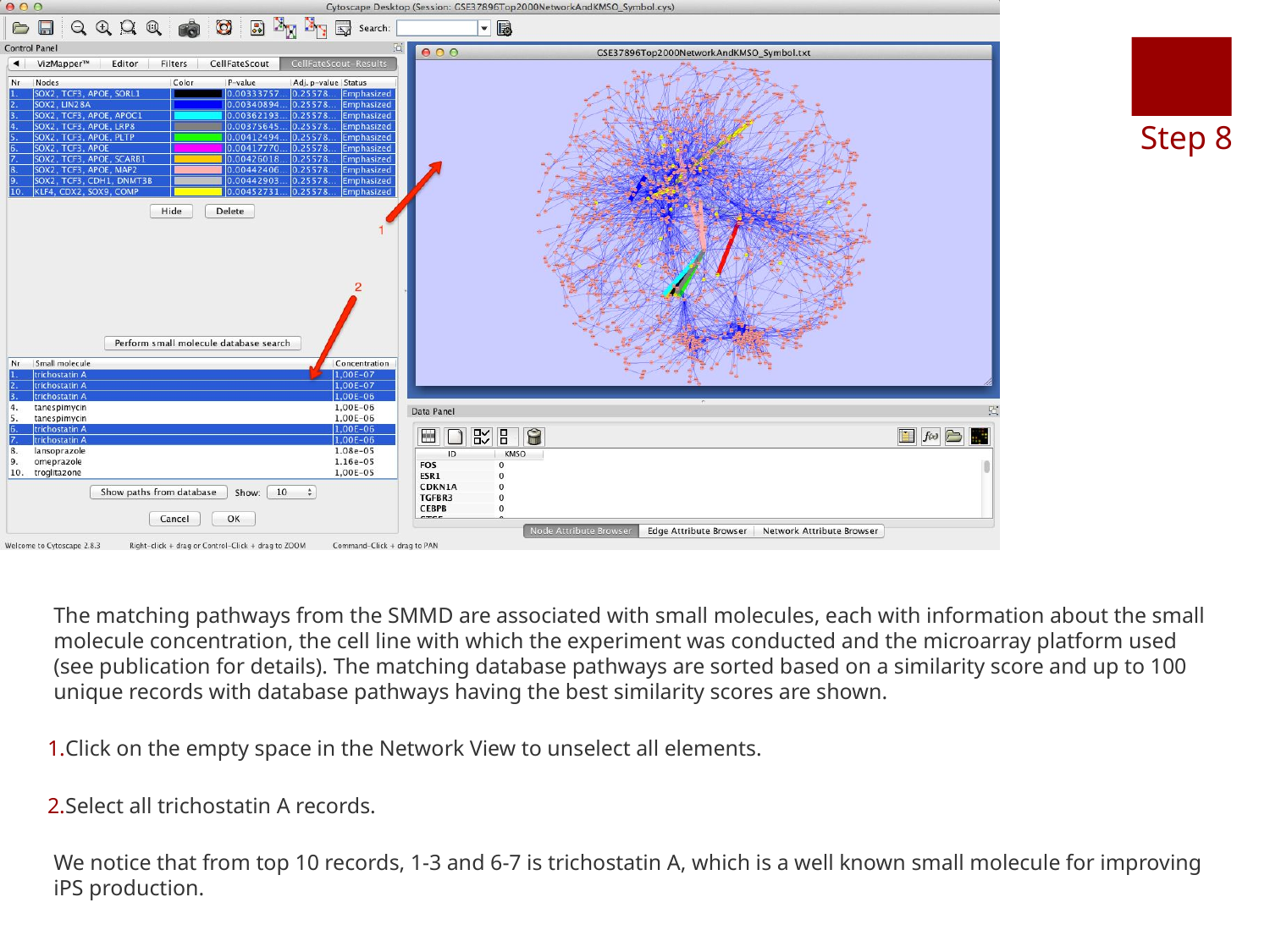

Step 8
The matching pathways from the SMMD are associated with small molecules, each with information about the small molecule concentration, the cell line with which the experiment was conducted and the microarray platform used (see publication for details). The matching database pathways are sorted based on a similarity score and up to 100 unique records with database pathways having the best similarity scores are shown.
Click on the empty space in the Network View to unselect all elements.
Select all trichostatin A records.
We notice that from top 10 records, 1-3 and 6-7 is trichostatin A, which is a well known small molecule for improving iPS production.

## Slide 11
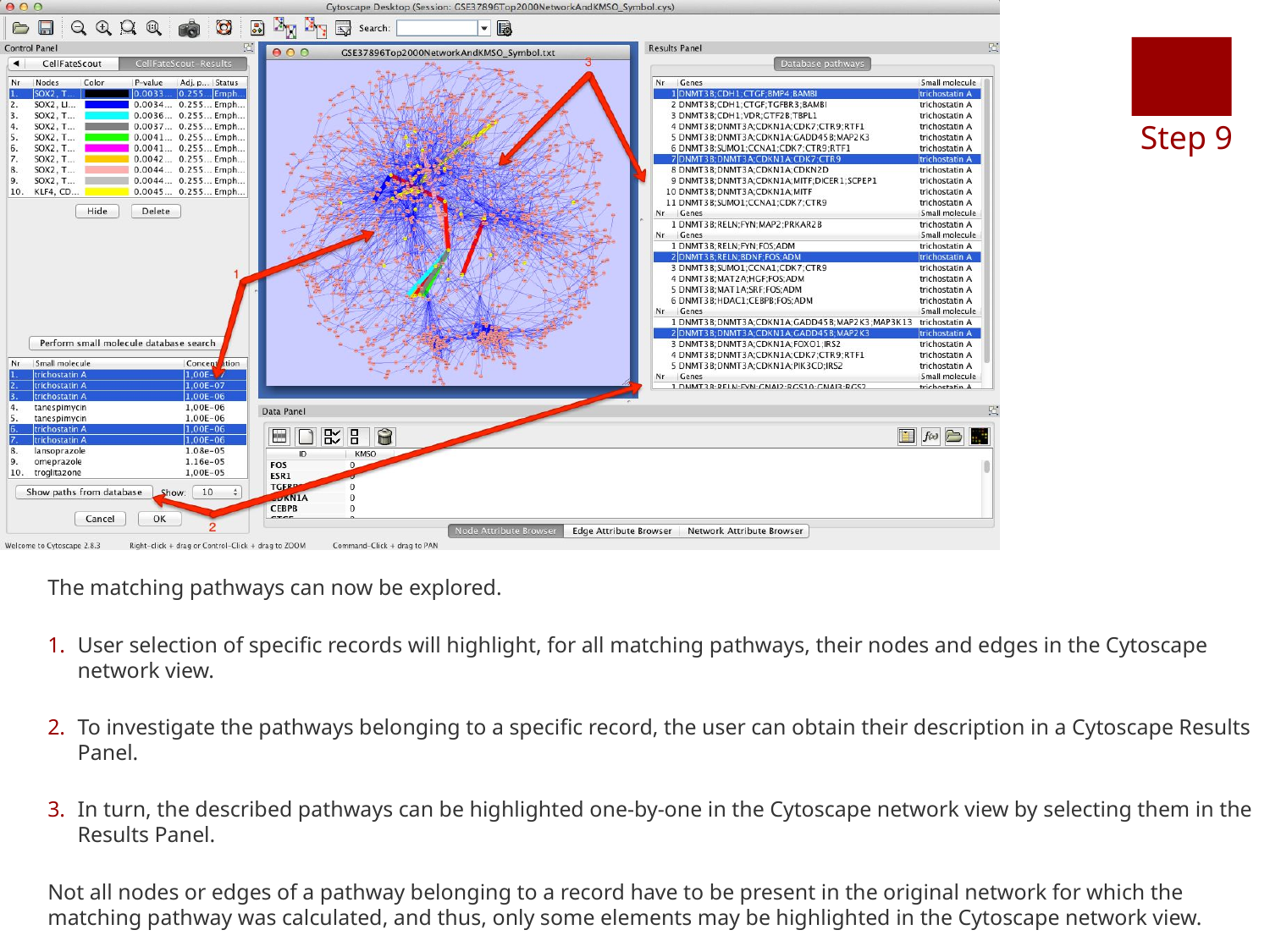

Step 9
The matching pathways can now be explored.
User selection of specific records will highlight, for all matching pathways, their nodes and edges in the Cytoscape network view.
To investigate the pathways belonging to a specific record, the user can obtain their description in a Cytoscape Results Panel.
In turn, the described pathways can be highlighted one-by-one in the Cytoscape network view by selecting them in the Results Panel.
Not all nodes or edges of a pathway belonging to a record have to be present in the original network for which the matching pathway was calculated, and thus, only some elements may be highlighted in the Cytoscape network view.
